# Breast osteoblast-like cells: a new biomarker for the management of breast cancer

**DOI:** 10.1038/s41416-018-0255-y

**Published:** 2018-10-17

**Authors:** Manuel Scimeca, Nicoletta Urbano, Rita Bonfiglio, Orazio Schillaci, Elena Bonanno

**Affiliations:** 1 Department of Biomedicine and Prevention, University of Rome “Tor Vergata”, Via Montpellier 1, Rome, 00133 Italy; 2 University of San Raffaele, Via di Val Cannuta 247, 00166 Rome, Italy; 3 OrchideaLab S.r.l, Via del Grecale 6, Morlupo, Rome, Italy; 4 Nuclear Medicine, Policlinico “Tor Vergata”, Rome, Italy; 5 Department of Experimental Medicine, University “Tor Vergata”, Via Montpellier 1, Rome, 00133 Italy; 6 IRCCS Neuromed, Pozzilli, Italy; 7 “Diagnostica Medica” and “Villa dei Platani”, Avellino, Italy

## Abstract

**Background:**

In this study, we investigated the relationship between the expression of the main in situ markers of breast cancer and the presence of breast osteoblast-like cells (BOLCs).

**Methods:**

We collected 100 breast biopsies. Serial paraffin sections were obtained from each biopsy to perform histological classifications and immunohistochemical analyses (RUNX2, RANKL, vimentin, TGFβ, Ki67, CD44, ER, PR and HER2).

**Results:**

Linear regression analysis showed a positive and significant correlation between the number of BOLCs and the expression of EMT-related markers (vimentin and TGFβ), Ki67 and ER. Conversely, we observed an inverse correlation between the number of CD44-positive breast cancer cells and the BOLCs. No significant differences were observed between the number of BOLCs and the HER2 scores.

**Conclusions:**

Morphological and molecular characterisation of BOLCs can lay the foundations towards understanding the biological basis of the formation of breast microcalcifications, and breast cancer metastasis to bone. The data here reported may be useful for the identification of breast lesions with high potential to develop bone metastasis.

## Introduction

In our previous study we demonstrated that the formation of osteoblast-like cells in breast tissues (BOLCs) is strictly related to the expression of the main epithelial-to-mesenchymal transition (EMT) markers.^1,2^ On note, BOLCs displayed functional affinity to osteoblasts since they produce calcified crystals made of hydroxyapatite^3^ that can be identified during mammographic exams as casting type calcification:^4^ a type of calcium deposits linked to neoductogenesis and poor prognosis. Also, we found that the presence of RUNX2 (Runt-related transcription factor 2)- and RANKL (receptor activator of nuclear factor κΒ ligand)-positive BOLCs in primary tumour correlated with the development of bone metastasis.^5^ Starting from these evidences, the aim of this study was to investigate the relationship between the expression of the main prognostic and predictive in situ markers of breast cancer and the presence of BOLCs at the primary lesion sites.

## Methods

We enrolled 100 patients from whom we collected one breast biopsy each (61.48 ± 1.44 years). Our study protocol was approved by independent ethical committee (reference number #94.13). From each biopsy, paraffin serial sections were obtained to perform histological classifications and immunohistochemical analyses.

### Histology

After fixation in 10% buffered formalin for 24 h, breast tissues were paraffin embedded. The 4-μm-thick sections were stained with haematoxylin–eosin (H&E).^6^

### Immunohistochemistry

We employed immunohistochemical techniques to study the BOLCs and the prognostic and predictive markers of breast cancer. Briefly, antigen retrieval was performed on 3-μm-thick paraffin sections using EDTA citrate pH 7.8 buffers for 30 min at 95 °C. Sections were then incubated for 1 h at room temperature with the following primary antibodies diluted 1:100: RUNX2 (clone 1D8, Novus Biologicals, USA), RANKL (clone 12A668, Abcam, Cambridge, UK) vimentin (clone 2D1, Novus Biologicals), TGFβ (clone 1D11.16.8, Ki67, Novus Biologicals), CD44 (clone 8E2F3, Novus Biologicals), ER (clone SP1, Novus Biologicals), and HER2 (clone 4B5, Ventana, Tucson, USA). RUNX2 (streptavidin-Texas-Red) and RANKL (streptavidin-FITC) were detected by using immunofluorescence technique. All the other reactions were revealed by HRP–DAB Detection Kit (UCS Diagnostic, Italy).^7^ Immunohistochemical positivity was evaluated on digital images (Iscan Coreo, Ventana, Tucson, AZ, USA) by a semi-quantitative approach. Specifically, immunoreactions for TGFβ, vimentin, CD44, ER and HER2 were evaluated by counting the number of positive breast-infiltrating cells (out of a total of 500 in randomly selected regions), whereas the evaluation of Ki67 expression was calculated in terms of percentage of positive cancer cells for Ki67 (out of a total of 500 in randomly selected regions). To assess the background of immunostaining we included a negative control for each reaction by incubating the sections with secondary antibodies (horseradish peroxidase (HRP)) and detection system (3,3-diaminobenzidine (DAB)). Reactions have been set-up by using specific control tissues as indicated in the data sheets.

### Statistical analysis

Linear regression analyses were performed to assess the correlation between the presence of BOLCs and the expression of vimentin, oestrogen receptor (ER), Ki67, CD44 and transforming growth factor-β (TGFβ) in breast cancer tissues. One-way analysis of variance was performed to assess the correlation between the presence of BOLCs and HER2.

## Results

**Fig. 1:**
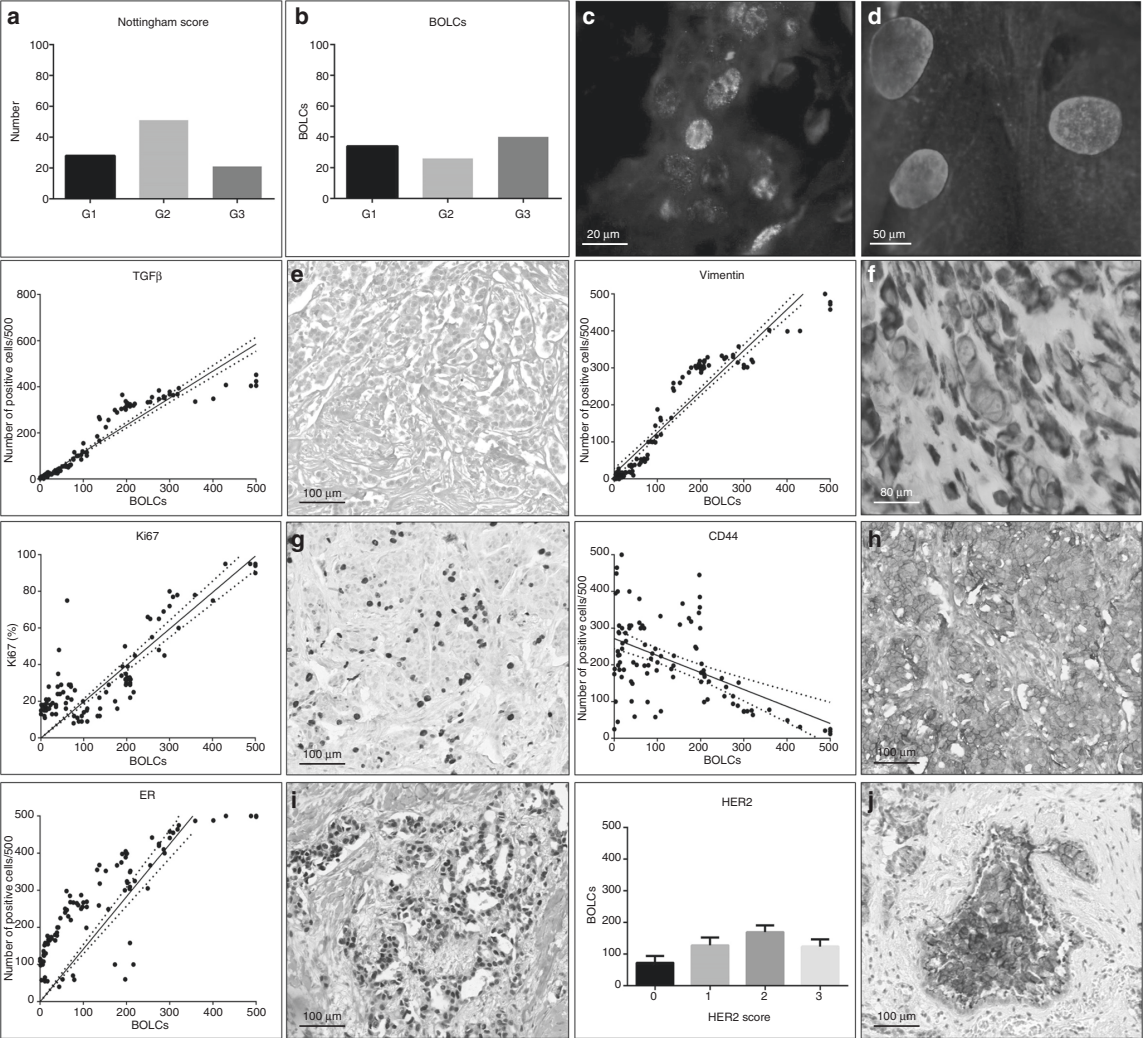
Breast osteoblast-like cells (BOLCs) and the expression of breast cancer biomarkers. **a** Graph shows the Nottingham histological score of breast-infiltrating carcinomas. **b** Graph displays the number of BOLCs in G1, G2 and G3 groups (Nottingham score). **c** Image shows BOLCs in infiltrating breast cancer; RANKL expression Texas-Red, RUNX2 expression FITC (scale bar represents 20 µm). **d** High magnification of BOLCs; RANKL expression Texas-Red, RUNX2 expression FITC (scale bar represents 50 µm). **e** Graph displays the correlation between BOLCs and the number of TGFβ-positive cells (*p* < 0.0001; *R*^2^ 0.865). Representative image of TGFβ cancer-positive cells in a breast-infiltrating carcinoma (scale bar represents 100 µm). **f** Graph shows the correlation between BOLCs and the number of vimentin-positive cells (*p* < 0.0001; *R*^2^0.810). Representative image of vimentin cancer-positive cells in a breast-infiltrating carcinoma (scale bar represents 80 µm). **g** Graph displays the correlation between BOLCs and the percentage of Ki67-positive cells (*p* < 0.0001, *R*^2^ 0.859). Representative image of Ki67 cancer-positive cells in a breast-infiltrating carcinoma (scale bar represents 100 µm). **h** Graph shows the correlation between BOLCs and the number of CD44-positive cells (*p* < 0.0001, *R*^2^ 0.627). Representative image of CD44 cancer-positive cells in a breast-infiltrating carcinoma (scale bar represents 100 µm). **i** Graph displays the correlation between BOLCs and the number of ER-positive cells (*p* < 0.0001, *R*^2^ 0.827). Representative image of ER cancer-positive cells in a breast-infiltrating carcinoma (scale bar represents 100 µm). **j** Graph shows the number of BOLCs in score 0, score 1, score 2 and score 3 groups (Her2 scoring system) (*p* = 0.581). Representative image of HER2 cancer-positive cells in a breast-infiltrating carcinoma (scale bar represents 100 µm)

The study of H&E sections allowed us to classify breast biopsies in ductal-infiltrating carcinomas according to Nottingham Histological system.^8^ Specifically, we observed 28/100 G1-infiltrating carcinomas (60.71 ± 2.28 years), 51/100 G2-infiltrating carcinomas (65.89 ± 2.87 years) and 21/100 G3-infiltrating carcinomas (58.36 ± 3.23 years) (Fig. 1a).

### BOLC detection

The presence of BOLCs in ductal-infiltrating carcinomas was detected by dual-colour immunofluorescence (Fig. 1b). Cells expressing simultaneously RUNX2 (the first transcription factor required for determination of the osteoblast lineage), and RANKL (a secreted molecule capable to regulate bone metabolism by activating the osteoclasts) were considered as BOLCs (Fig. 1c, d). Specifically, we evaluated the number of BOLCs out of a total of 500 breast cancer cells analysed.

### Prognostic and predictive biomarkers vs BOLCs

Immunohistochemical analysis allowed us to evaluate the expression of vimentin, ER, Ki67, CD44 and TGFβ with respect to the presence of BOLCs in breast-infiltrating carcinoma.

Our results showed a positive and significant correlation between the number of BOLCs and the expression of both EMT-related markers, TGFβ and vimentin (TGFβ *p* < 0.0001, *R*^2^ 0.865; vimentin *p* < 0.0001, *R*^2^ 0.810) (Fig. 1e, f). We also noted a positive correlation between proliferation index of breast-infiltrating carcinomas, evaluated in terms of percentage of Ki67-positive cells and the number of BOLCs (*p* < 0.0001, *R*^2^ 0.859) (Fig. 1g). Conversely, we observed an inverse correlation between the number of CD44-positive breast cancer cells and the BOLCs (*p* < 0.0001, *R*^2^ 0.627) (Fig. 1h). Noteworthy, this inverse correlation was particularly relevant in those breast-infiltrating carcinomas characterised by a high number of BOLCs (>200/500) (Fig. 1h). We found a linear increase between ER-positive breast cancers cells in tissues and the amount of BOLCs (Fig. 1i). Specifically, linear regression analysis showed a significant positive association between ER and BOLCs (*p* < 0.0001, *R*^2^ 0.827) (Fig. 1i). Finally, no significant differences were observed between the number of BOLCs and the HER2 scores (*p* = 0.581) (Fig. 1j).

## Discussion

In the research field about breast cancer metastasis to bone, BOLCs can represent a new reliable biomarker for both early detection of metastatic process and the development of new drugs. Nevertheless, no studies were performed about the histochemical/histological characteristics of breast lesions containing BOLCs. Therefore, here we investigate 100 breast-infiltrating carcinomas to study the correlation between the presence of BOLCs at the primary lesion sites and the in situ expression of the main prognostic and predictive biomarkers of breast cancer. As expected, we observed a significant increase of TGFβ and vimentin expression in breast cancer tissues with higher amount of BOLCs. Indeed, as previously demonstrated, the EMT phenomenon represents the biological “substrate” to BOLC generation.^1^ Also, coherently with the hypothesis that the BOLCs are responsible for bone metastasis formation,^5^ we observed a significant positive association between the percentage of Ki67-positive breast cancer cells and the presence of BOLCs. Surprisingly, the analysis of CD44 indicates an inverse correlation with BOLCs. Surprisingly, the analysis of CD44 indicates an inverse correlation with BOLCs. In recent years, several CD44 isoforms have garnered significant attention because of their utility as breast cancer stem cell markers;^9^ in a study of Jeong et al.,^10^ the authors use the anti-CD44 antibody (8E2F3) to demonstrate that a breast cancer stem cell phenotype (CD44+/CD24−) was significantly associated with the basal-like molecular subtype, which is predictive of a poor prognosis. Therefore, BOLC generation and cancer stem cell development and maintenance seem to be opposing phenomena in breast cancer occurrence and progression. Noteworthy, in line with the literature, we also observed a high number of BOLCs in ER-positive breast carcinomas. From the molecular point of view, the hyper-activation of ER signalling may explain the formation of BOLCs and the consequent bone metastasis development. In fact, it is known that both oestrogens sustain osteoblast activity^11^ and ER-positive tumours preferentially spread to bone.^12^ Conversely, the deregulation of HER2 expression do not seem to have any particular involvement in BOLC formation/activity.

## Conclusion

The morphological and molecular characterisation of BOLCs can lay the foundations for the comprehension of the biological basis of the formation of breast microcalcifications and breast cancer metastasis to bone.

In addition, data here reported can be used for the identification of breast lesions with high potential to develop bone metastasis. In this context, we can speculate that 99mTc sestamibi high-resolution single-photon emission computed tomography could be a suitable approach for the detection of breast cancer lesions characterised by high percentage of BOLCs. Distribution and pharmacokinetics of 99mTc sestamibi appears particularly suitable for the in vivo detection of BOLCs due to its propensity to accumulate into mitochondria. Indeed, ultrastructural investigations displayed the presence of numerous mitochondria in both BOLCs and osteoblasts during calcification production. In line with these evidences, our preliminary results showed a significant increase of sestamibi uptake in breast cancers lesions with a high number of BOLCs (>200/500) as compared to cancer lesions with no/rare BOLCs.
